# Editorial: Substance abuse and the microbiome

**DOI:** 10.3389/adar.2024.12734

**Published:** 2024-03-06

**Authors:** Prakash Nagarkatti, Mitzi Nagarkatti, Shilpa Buch

**Affiliations:** ^1^ Department of Pathology, Microbiology and Immunology, School of Medicine, University of South Carolina, Columbia, SC, United States; ^2^ Department of Pharmacology and Experimental Neuroscience, University of Nebraska Medical Center, Omaha, NE, United States

**Keywords:** drug abuse, microbiota, cannabinoids, opioids, dysbiosis

The gastrointestinal (GI) tract harbors a highly complex array of microorganisms that play a critical role in regulating homeostasis in the body. Thus, any perturbations or imbalance, termed dysbiosis, can trigger disease. While the direct effects of abused substances on various organ systems such as the brain, have been well-recognized, whether drug misuse can also lead to dysbiosis, resulting in clinical disorders remains an interesting possibility that is actively researched. This Special Issue was therefore focused on how substance use disorders (SUD) are linked to dysbiosis, and how altered gut microbial composition can influence the functions of the Central Nervous System (CNS) and the immune system. It was also the goal of this Special Issue to explore whether the stabilization of gut microbiota could lead to the restoration of clinical disorders that are triggered by drugs of abuse.

In this Special Topic Issue, we present four articles: In the first article, Varsha et al. describe how the ability of cannabinoids to suppress inflammation could likely be mediated, in part, through the alterations in the gut microbiome and metabolome. There is significant evidence demonstrating that cannabinoids such as delta-9-tetrahydrocannabinol (THC), and Cannabidiol (CBD) act as anti-inflammatory agents. Also, these cannabinoids have been approved by the FDA to treat several clinical disorders. For example, THC has been approved by the FDA to treat HIV/AIDS-induced anorexia as well as chemotherapy-induced nausea and vomiting in cancer patients undergoing chemotherapy. CBD has also been approved by the FDA to treat certain types of epilepsy syndromes. Moreover, several states in the US have legalized cannabis for medicinal and/or recreational use. Thus, it is important to understand whether the anti-inflammatory effects of cannabinoids are mediated via the regulation of dysbiosis and, if so, the impact of this on health. This review captures the mechanism(s) that trigger dysbiosis following exposure to cannabinoids. Additionally, it highlights how cannabinoids can induce microbial secondary bile acids, short-chain fatty acids (SCFA), and indole metabolites, that can have an immunoregulatory role even in distant organs.

The second review by Ellermann is closely related to the first article in that the review focuses on endocannabinoids. An important highlight of this article is the demonstration that changes in the gut microbiome caused by external factors such as diet or disease can have a significant impact on the endocannabinoid tone. This is critical inasmuch as endocannabinoids regulate a wide array of important bodily functions such as memory, sleep, temperature control, pain control, appetite, and immune functions. The article also highlights endocannabinoid-mediated regulation of naturally occuring bacteria within the gut microbiome. Additional exciting areas covered by this article include the preclinical studies demonstrating that engineered gut bacteria synthesizing the host N-acylethanolamides could be potentially used to treat diseases that involve aberrant lipid signaling, including obesity and inflammatory bowel diseases.

Drug abuse by HIV-1/AIDS patients has been shown to increase viral load and accelerate the disease progression. The third article by Ray et al. highlights the evidence that the gut microbiome plays an important role in the pathogenesis of HIV-1-linked drug abuse and subsequent neuroinflammation and neurodegeneration. It is well documented that drug abuse can disrupt the gut-brain axis resulting in dysbiosis, and altered expression of neurotransmitters, bile acids, and metabolites, including SCFA. Such alterations can activate a wide range of pro-inflammatory signaling pathways, which, in turn, can impact the CNS through the hypothalamic-pituitary axis, ultimately resulting in pain, stress, and anxiety. The article highlights how understanding the mechanism(s) underlying how drugs of abuse alter the microbiota in HIV-1/AIDS patients could aid in the development of better treatment modalities for drug abuse-related disorders.

The fourth article by Herlihy and Roy focuses on opioid-mediated microbial dysbiosis and its impact on behavior. The review highlights how drug-induced dysbiosis can lead to an increased prevalence of pathogenic bacteria, in turn, manifesting as a compromised gut barrier with consequent systemic translocation of bacteria that trigger proinflammatory cytokine release. The microbiome also communicates with the brain by sending signals through the vagus nerve. The article also discusses how the microbiome can increase microglial activation in the brain as well as dysregulation of brain-derived neurotrophic factor (BDNF) signaling during drug use. All such alterations in the microbiome also impact the behavioral consequences of drug use. Together, these studies suggest that preventing dysbiosis could likely attenuate behavioral symptoms associated with drug use.

In summary, this Special Issue consisting of four review articles comprehensively discusses the complex interactions between drug use and microbial dysbiosis. It also highlights how dysbiosis is closely associated with the endocannabinoid and immune system while communicating with the brain through the gut-brain axis, thereby regulating pain, anxiety, and behavior. The articles highlight the challenges and opportunities to advance this research to better understand and control drug use and behavioral disorders.

